# Etiopathogenesis of Burkitt's lymphoma: a lesson from a BL-like in CD1 mouse immune to Plasmodium yoelii yoelii

**DOI:** 10.1186/1750-9378-6-10

**Published:** 2011-07-09

**Authors:** Filiberto Malagon, Jorge Gonzalez-Angulo, Elba Carrasco, Lilia Robert

**Affiliations:** 1 Departamento de Microbiología y Parasitología, Laboratorio de Malariologia Facultad de Medicina, Universidad Nacional Autónoma de México. Cd. Universitaria, México, D.F., México; 2 Facultad de Estudios Profesionales, Iztacala, Universidad Nacional Autónoma de México. Iztacala, Edo. de México, México

## Abstract

**Introduction:**

There is a jaw cancer that develops in children five to eight years old in holoendemic malaria regions of Africa, associated to malaria and Epstein Barr virus infections (EBV). This malignancy is known as endemic Burkitt's lymphoma, and histopatologically is characterized by a starry sky appearance. To date, no histopathologic expression of Burkitt's lymphoma has been reported in non-genetically manipulated experimental animals. The purpose of the study is to describe the case of a mouse immune to *Plasmodium yoelii yoelii *(Pyy) that developed a Burkitt's lymphoma-like neoplasm after repeated malaria infections.

**Results:**

Immune mouse 10 (IM-10) developed neoplasms at eight months of age, after receiving three Pyy inoculations. At autopsy eight subcutaneous tumors were found of which the right iliac fosse tumor perforated the abdominal wall and invaded the colon. The histopathologic study showed that all neoplasms were malignant lymphomas of large non-cleaved cells also compatible with variants or previous states of development of a Burkitt's lymphoma-like. The thymus, however, showed a typical starry sky Burkitt's lymphoma-like neoplasm.

**Conclusions:**

Neoplasm development in CD1 mouse is associated to both, immunity against malaria and continuous antigenic stimulation with living parasites.

It is the first observation of a histopathologically expressed Human Burkitt's lymphoma-like neoplasm in a non-genetically manipulated mouse.

Chronic immune response associated to neoplasms development could probably be not an exclusive expression of malaria-host interaction but, it could be a pattern that can bee applied also to other agent-host interactions such as host-bacteria, fungus, virus and other parasites.

## Introduction

Malaria in the tropics is a problem of worldwide concern, because of the millions of persons falling ill and dead each year [1] and, the children suffering of Burkitt's lymphoma have to be counted among them. Dr. Denis Burkitt first described this neoplasm in Ugandan children in 1957 [2]. Today this tumor is known as Burkitt's lymphoma (BL) which corresponds to a neoplasia of B cells that grows mainly in the jaw, and some times also in ovary and testis. Its major incidence occurs in African children five to eight years old in a geographical band of latitude 10 degrees north and 10 degrees south the equator, also called the Burkitt's lymphoma belt or the malaria belt, because it coincide with holoendemic malarious territories i.e. with areas of the highest malaria transmission intensity. Three BL types are distinguished, the endemic form, the sporadic and the associated to HIV infections. The endemic type occurs in Africa holoendemic malaria territories, the sporadic elsewhere out of Africa and the associated to HIV infection is everywhere Africa included [3].

Just after BL discovery it was clear to Dalldorf [4] that the geographical association between malaria and the BL incriminated in some way the malaria parasites with the neoplasia development, although there were no arguments to substantiate this idea. Few years later Antony Epstein in collaboration with Yvonne Barr and Bert Achong described the presence of a virus in the neoplastic cells of Burkitt's lymphoma [5], which is known since then as Epstein Barr virus (EBV). The discovery of the virus opened controversial opinions between those that favor malaria parasites or EBV, as implicated in the malignant transformation of B lymphocytes. Arguments favoring one or the other microorganism has come and gone but still today discussion has not reach an end.

## Methods

### Mice

CD1 male mice of 20-25 g body weight were used as experimental animals and for Pyy strain maintenance. Certified CD1 Charles River strain was used, under the guidelines of the Faculty of Medicine Ethics Commission and the Mexican Official Norm NOM-062-ZOO-1999 on Technical Specifications for Breeding, Care and Uses of Laboratory Animals.

### Parasite

The lethal strain of *Plasmodium yoelii yoelii *(Pyy) was used and maintained by mouse-to-mouse passages. For maintenance, 10 μL of Pyy infected blood was poured into 5 ml of saline and 0.5 ml of this suspension were inoculated intraperitoneally to each mouse

### Immune mouse

For a mouse to be regarded in a status of sterile immunity it has to be able to overcome a primary Pyy infection that is usually lethal to non-immune mice, without therapy intervention. The mouse has to resist a Challenge after a month's survival to the primary infection and its blood has to be no infective to non- immune mice, after a week of the challenge.

### Epstein Barr virus (EBV)search

The presence of EBV in the tumor as well as in the animal's blood, was studied by two procedures: Polimerase chain reaction (PCR) and Electron microscopy (EM). For EM, a tumor sample was processed: first, it was fixed with 2.5% glutaraldehide mixed with sodium cacodylate 0.1 M, and then post fixed with 1% osmium tetra oxide and dehydrated in ethyl alcohol concentrations from 50%, 70%, 80%, 90% and 100%. Then the fragment was treated with propylene oxide for 30 min and then mixed with Epon, and included overnight. After that, the fragment was cut and the specimen supported on a 30 μm mesh and stained with 4% uranyl acetate and 0.35% lead acetate, according to Glavert [6]. The specimens were observed in a Jeol electron microscope JEM-1200 Ex II.

For PCR analysis, DNA was extracted and purified from another fragment of the tumor and a blood sample, using the chloroform method. Whole DNA of the mouse's blood and tumor samples and an EBV DNA control sample were tested by PCR to search for EBV in an assay with primers specific for the EBNA1 gene:

5-TCATCATCATCCGGGTCTCC-3 and 5-CCTACAGGGTGGAAAAATGGC-3 [7].

## The CD1 Burkitt's lymphoma model

Nowadays, the experimental study of BL is limited by the lack of an animal model to reproduce and study the disease. Hori M [8] studied more than 30,000 cases of lymphomas in NFS.V+ mice and 400 in AKXD mice, without finding histological features comparable to human BL. According to Kovalchuk [9], tumors with histological and phenotypic features of BL have never been described in mice. However, he experimentally reproduced BL in the mice, although his mice had to be humanized by genetic manipulations to enable them to express BL.

These experiments were done in CD1 male mice and their first malaria infection was induced at about two months of age. Due to the lethality of the Pyy strain, the first infection decided the fate of mice that can only be death or survival. As a rule, most of them died, but exceptionally some of them survive. If they survive they acquire a sterile immune state. When parasites enter again, a temporal infection with very low parasitemia is established; usually reaching in its peak no more than 2%, and thereafter the parasites disappear at variable lapses of time, from three to twelve days with a mean of 5.5 days. After this time no parasites are found by blood microscopy or sub-inoculation of blood or organs into non-immune mice.

We observed that those few CD1 mice that naturally survived and acquired sterile immunity against the lethal strain of *Plasmodium yoelii yoelii *(Pyy) were in risk of developing a neoplasm, if antigenic stimulation (re-infections) with living parasites was repeated several times. We observed a CD1 mouse immune to Pyy that developed eight subcutaneous and one peritoneal lymphocytic lymphomas (also compatible with variant of Burkitt's lymphoma) and one more in the thymus with the histopathologic features of a human Burkitt's Lymphoma-like neoplasm. This mouse identified as immune mouse 10 (IM-10) was part of a group of 13 immune mice which developed different types of neoplasia during these experiments, and whose entire observations will be published elsewhere.

## Neoplasm developed by CD1 immune mouse 10

### The immune mouse 10

**Figure 1:**
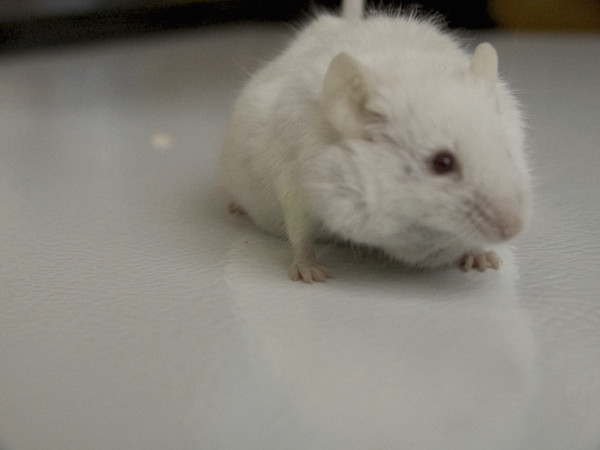
**Immune Mouse 10, before autopsy**. See the tumor running along the front neck that somewhat deform the face.

On third October 2005 an experiment was started in which 22 mice were inoculated by the intra-peritoneal route with a cultivated strain of Pyy, on an infectivity test. After one month's observation, blood infection by the cultivated Pyy was not detected in any of the mice. In order to see the response of these mice to the virulent parasite strain from which the cultivated forms arose, they were inoculated with Pyy infected blood and the infection was followed. Ten days later, all mice had died except two of them. On December 13^th ^both surviving mice were challenge with Pyy infected blood. Parasites were not detected in their blood after the third day and, their blood was not infective by transfer to non-immune mice after the fourth day, thus both mice survived and were qualified as immune. One of these mice was the immune mouse 10 (IM-10). On the 23rd of February (2006) IM-10 was inoculated with Pyy infected blood when participating in a Pyy phagocytosis experiment. On June 12^th ^a neoplasia was detected in the abdomen of IM-10. On 26^th ^June the neoplasia had grown up and a new tumor was detected in the neck. On 15^th ^August IM-10, another neoplasm of the neck was detected up the sternum measuring about two and a half cm long, apparently conformed by a single movable mass (see Figure 1). The abdominal neoplasm was also movable but adhered to deep tissues, and another two neoplastic bodies grew in both iliac fosses, however, IM-10 looked not much affected. Blood samples stained with Giemsa and sub inoculation of its blood into non-immune mice, showed that this mouse was free of malaria infection. On October 14^th ^, as IM-10 was adynamic, and his facial and shoulder's hair was falling it was decided that it participated in a last phagocytosis experiment before it was autopsied. The experiment consisted on inoculation of Pyy infected blood to IM-10 by intra-peritoneal route, leaving it to rest during four days and then (October 18^th^) its blood was transferred to two recipient mice to detect infection and, then it was inoculated with Pyy infected blood again, leave for one hour and then intra-peritoneal exudates were collected under anesthesia, to check for phagocytosis of malaria parasites by macrophages, thereafter IM-10 was sacrificed by an overdose of anesthesia and autopsied.

### Autopsy

**Figure 2:**
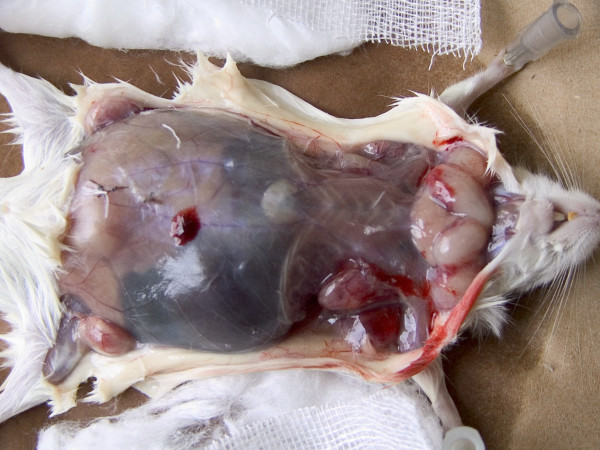
**Immune Mouse 10, first plane autopsy**. It shows four tumoral masses along front neck, two on each side, covering most of the thyroid gland of which just a portion is seen at the center. Another four neoplastic bodies are seen distributed on right and left axilla and right and left iliac fosse. Low central abdomen shows another mass behind the abdominal wall.

**Figure 3:**
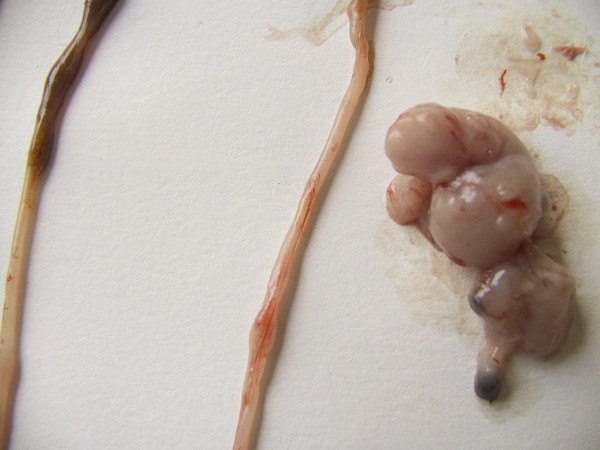
**Immune Mouse 10, second plane authopsy**. It shows the abdominal mass neoplasm fussed with the colon, which can be identified by the feces dark spots.

eight neoplastic masses were observed (Figure 2), four of them located around the maxilla and the front neck, one on each axilla and one on each iliac fossa. The tumors in the neck were adhered to the surface of the thyroid gland. Each single tumor was between 1.7 and 1.8 cm long, profusely vascularized. The four neoplastic masses of the neck were covered together by a single transparent membrane, with adherence at some points to muscle and skin, branched blood vessels joined the tumors with the thyroid gland. The subcutaneous neoplasms at both iliac fossa measure 1.5 cm long and both were adhered to the skin and well vascularized. The one on the right perforated the abdominal wall and was seen fussed to a portion of the colon, it measured 6.5 cm in its whole length (Figure 3) and was not vascularized, it was the tumor localized in the abdomen. Both iliac fossa tumors were well vascularized and encapsulated. Both axilla tumors were also encapsulated and with no apparent invasion to other tissues. Liver, spleen and thymus were hypertrophied, kidneys with no macroscopic pathology, suprarenal glands were rough on their surface and augmented in size, brain, heart and lungs were macroscopically normal.

## Histopathology

### Organs with no proliferative lesions

#### Lung

It shows pneumonitis with inflammatory or neoplastic infiltrate surrounding the veins and bronchioles. Arteries not seemed to be affected.

#### Spleen

Congested, showing multi-nucleated gigantic cells containing malaria pigment, and big nuclei containing chromatin grains. Nuclei were apparently non-neoplastic.

#### Brain

Signs of hypoxia, increased population of lymphocytes in the meningeal layer, without signs of meningitis, with no malaria pigment nor lesions in vessels, mostly normal.

### Organs with proliferative lesions

#### Left iliac fossa's neoplastic tissue

**Figure 4:**
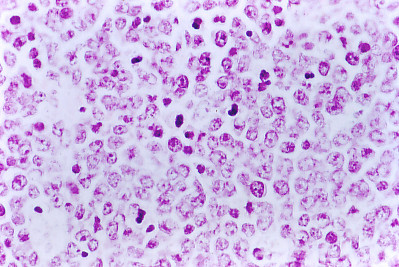
**Immune mouse 10, left iliac fosse neoplasic tissue**. Histopathology. Hyper-chromatic lymphoid cells that correspond to cells under mitoses processes of a malignant lymphocytic lymphoma poorly differentiated. Hematoxylin & Eosin (× 40).

Hyper-chromatic lymphoid cells with medium size cytoplasm and open nuclei with coarse chromatin and some cells showing one or two prominent nucleoli, six to seven mitosis (shown at × 40 magnification) that suggest cells under mitoses processes of a malignant lymphoma of large non cleaved cells (Figure 4).

#### Colon neoplastic tissue

**Figure 5:**
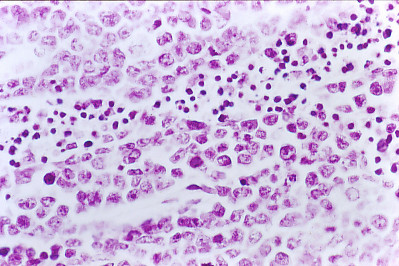
**Immune mouse 10, colon fussed neoplasic tissue**. Histopathology. Flux of necrotic and pycnotic cells running inside a vein whit no erythrocytes visible. H&E (× 40).

A uniform cell layer was found with very similar cellular composition to those found in the above lesion, but also presented an influx of lymphoma cells and other white cells running in the lumen of a blood vessel (Figure 5). Malignant lymphoma of large non cleaved cells.

#### Kidney

The tissue surrounding renal tubules was infiltrated by lymphoma cells. This process was focalized.

#### Thymus

**Figure 6:**
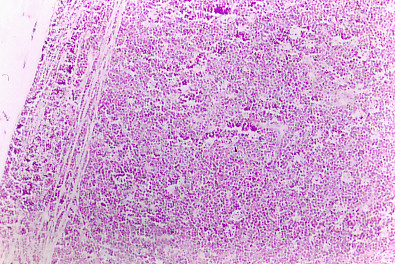
**Immune mouse 10, thymus tissue, Histopathology**. Normal tissue is rejected by the tumor. Neoplastic tissue shows a very typical starry sky, characteristic of human burkitt's lymphoma. H&E (× 10).

**Figure 7:**
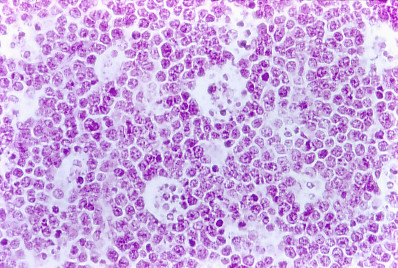
**Immune mouse 10, thymus tissue**. Histopathology. Typical image of a starry sky as seen in human Burkitt's lymphoma (× 40).

Neoplastic tissue was rejecting normal thymus tissue (Figure 6). The neoplastic tissue shows a very typical starry sky characteristic of human Burkitt's lymphoma cells seem uniform with very small variations in size and form, and very little cytoplasm, with round central nuclei, of well define borders, with coarse chromatin and with one or two basophile nucleoli (small non cleaved cells), it call our attention the normal isolated macrophages with phagocyted cellular debris in its cytoplasm that conforms the islands of the starry sky (for more detail see Figure 7).

From all tumors and organs observed, just the thymus presented the characteristic morphology of the human Burkitt's lymphoma-like neoplasm. All other tumors had features that suggest lesions of malignant lymphoma of large non cleaved cells, also compatible with variants or previous states of development of a Burkitt's lymphoma-like.

#### Epstein Barr virus search

The PCR analysis detected EBV in the control sample but did not detect EBV DNA in the tumor, nor either in the blood. Electron microscopy did not detect EBV particles and not any other particle suspicious to be of viral origin.

#### Blood parasites Search

The blood taken from IM-10 four days before the last inoculation of Pyy did not contained malaria parasites, as demonstrated by the non-infectivity of this blood to non-immune mice.

## Malaria, Epstein Barr Virus infection and Burkitt's lymphoma natural history in African Children

In order to explain our views about the emergence of malignant cells in mouse's tissues, which acquire the histological features of a BL and its correlation with the human BL, a remembrance of the human life cycle of people living in the holoendemic malaria regions of Africa, and their association with malaria and EBV infection, seems to be necessary.

A baby borne in Africa holoendemic malaria regions is coming to a world in which she or he has to fight to survive to malaria infections. If these babies are born free of malaria infection, their prime malaria infection is mostly experienced during the first year of life, usually after the first six months of age and from here on they are exposed to repeated infections for the rest of their lives. In some countries they receive an average of one infected bite each 3.6 days all year round [10], while in others 25 infective bites per month are expected [11]. In this mosquito biting atmosphere, *P. falciparum *morbidity reach a peak at around the age of five, leveling off after 10 years old [12]. Children thus live two stages in their life (in relation to malaria), one between the ages of one to five, in which they build an immune state to be protected, no against malaria infection but against clinical malaria, so this is a pre-immune stage. The second stage comprehend ages from 5 to 10 in which most surviving children have already acquired a state of clinical immunity protection [13] so it is an immune stage, although protective immunity to malaria infection would never be acquired [14].

EBV lives in the B lymphocytes of 95% of human beings whatever they reside and, in 100% of holoendemic malaria residents in Africa. Primary infection by EBV is acquired at different ages in Africa, the babies first infection may be acquired when passing through the vagina during delivery [15], or in the firsts months of life through salivary transmission, with minor or no clinical signs. Primary EBV infection induces cellular and humoral responses, which control the infection but do not eliminate it, passing to a latent state during all their lives [16].

Although children that survive and reach the second stage will continue being infected by malaria parasites and keep EBV latent infections for all their life, they do not usually die from any of these two infections, however, they are exposed to develop other diseases in which the immune system is involved as result of their continuous antigenic stimulation, such as the nephrotic syndrome [17], hyperreactive malaria splenomegaly [18], as well as the same BL. This is suggestive that possibly some other diseases linked with the continuous activation of the immune system by the malaria parasites are still to be recognized.

To better understand the elements that precede the emergence of Burkitt's lymphoma of humans and Burkitt's lymphoma-like in the rodents, we produce a list of some of these elements that may be compared to make some light, as follows:

1) BL and BL-like neoplasm of the mouse are associated to their specific malaria parasite infection, *Plasmodium falciparum *in humans and *P. yoelii yoelii *in the mouse.

2) *P. falciparum *infection in humans, and *P. yoelii *in the mouse have two or more possible outcomes. Whereas in the mouse the outcomes can be death or sterile immunity, in humans the outcomes can be death, clinical immunity and some others immunity linked pathologies.

3) In humans just about 1% of those exposed to infections *by P. falciparum *die, whereas 99% acquire clinical immunity. In contrast, most infected mice die from the infection and just about 0.2% acquires sterile immunity.

4) In humans immunity is reached after about five long years of exposure to transmission of the parasites, while in the mice immunity or death is defined with the first infection, in about a month in this experiments.

5) Endemic BL of humans and BL-like neoplasm of the mouse, both are linked to an immune response against their own malaria parasites.

6) Endemic BL in humans appear once an immune status against *P. falciparum *is established, after years of continuous antigenic stimulation, and the stimulation persist, while in the mouse it appears after a sterile immunity has been developed and the antigenic stimulus persists.

7) In humans the endemic BL is accompanied by *P. falciparum *infection and almost invariably by EBV infection, while in the mouse BL-like the infection by *P. yoelii *is not accompanied by EBV infection because this virus does not infect laboratory rodents nor has this ever been reported in these animals [19], see table 1.

**Table 1:** Comparison between endemic human Burkitt' lymphoma and Burkitt's lymphoma-like of the mouse.

	Endemic BL	BL-like
host species	Humans	CD1 mice

infecting Plasmodium	P. falciparum	P. yoelii yoelii

outcome of infection	death orclinical immunity	death orsterile immunity

population survival	99%	0.2%

time to reach immunity	about five years	about one month

immune state necessary	Yes	yes

virus association	Epstein-Barr virus	not known

The view of these elements suggest that three components have to be considered when thinking on the neoplasm genesis: the malaria parasite, the EBV and the immune response of the host, persistently activated by both malaria parasites and EBV, and possibly by other agents.

BL is a neoplasia of B cells and all Burkitt lymphoma cells invariably present translocation of c-myc/IgH genes as a landmark character, independently of the BL type to which it belongs. Therefore, for B cells to be transformed into malignancy the c-myc genes have to be activated and, for this to happen, translocation of c-myc/IgH has to take place. It becomes clear that it is of the uppermost importance to define what causes the translocation in the B cells. Most people working on the field would incriminate EBV as the cause of translocations [20,21] and, many others would involve the association of malaria infection to the emergence of BL, and to the translocation of c-myc. However, there are no definite evidences that one or the other agent is the direct cause of c-myc translocation [22,23]. Furthermore BL may exist in absence of malaria, and EBV infections [24,25], therefore, if Burkitt's lymphoma exist in absence of malaria and EBV infection, translocation of c-myc/IgH is not carried out by any of these two agents (as it occurs in most sporadic Burkitt's lymphoma), then If we discard both transmissible agents, as direct cause of c-myc translocation, just immunity, would explain the genesis of BL. Although malaria parasites and EBV are playing a very important role for the B cells to be transformed, they are not "per se" the direct cause of translocation, another factor involved is necessary and this factor may well be the host himself through his immune response.

## Translocation of c-myc/IgH-Burkitt's lymphoma as autoimmune-disease

Regarding the immune response as the central element in translocation, then BL can develop in absence of malaria parasites and EBV, and both could be supplanted for other agents either chemical or biological, that chronically activate the host immune response. The facts available today would incriminate malaria parasites and EBV as the biological agents involved in endemic BL, as the constantly repeated antigen stimulating factors of the immune response, that seems to lead to c-myc translocation, B cells malignization and genesis of Burkitt's lymphoma.

In principle, it would appear difficult to sustain that the host immune system is the cause of neoplasm genesis through its own cellular components, however, there is a story of at least 33 years that links immunity to malignancy, which probably started when Philip J. Fialkow [26] realized that immunologic mechanisms induce chromosomal aberrations which in turn play a role in the genesis of malignancy, additionally he noticed that increased frequency of neoplasms were associated to a variety of autoimmune disorders, e.g. autoimmune hemolytic anemia and lymphoid neoplasia. Experiments of the time indicated that lymphoid neoplasms arise with considerable frequency in animals injected with immunologically competent foreign lymphoid cells. He declared that autoimmune disease and the neoplasm may both be manifestations of the same process, and he cited Dameshek, who refers to the autoimmune disease and neoplasm as immunoproliferative disorders, suggesting that the only essential difference between the autoimmune disease and the malignancy is the greatly expanded cell mass in the latter.

Current concepts to reconstruct the processes that lead to an efficient adaptive immune response, sustain that the antigenic stimulation of naive B cells triggers a refined mechanism to accomplish the hallmark of adaptive immunity, characterized by its delicate specificity for foreign antigens, leading to the secondary diversification of the immunoglobulin genes through class switch recombination (CSR) and somatic hyper mutation (SHM) which are initiated by activation-induced cytidine deaminase (AID) [27]. Once a B cell responds to the antigen stimulation, before becoming an antibody producing plasma cell, some rearrangements and sequence modifications in the Ig genes are needed in order to be able to produce an isotype of antigen receptor that perfectly matches the recognized antigen. These processes are done through CSR or SHM and in both it is initiated by the enzyme AID mainly produced by the B cell germinal centers. This enzyme generates DNA double strand breaks, which needs to be repaired to tailor the gene to codify a protein that precisely matches the antigen recognized. At the end of the process the new tailored B cell become a clone of memory B cells that will reproduce when stimulated by the same antigen. To the B cell, these processes are normal in the way of establishing an immune response and, occur each time a naïve B cell recognizes a new antigen. When AID mediated lesions are improperly repaired it can promote chromosomal translocations of which the most studied is the c-myc/IgH t(8;14) that deregulates c-Myc giving rise to neoplastic cells in human BL [28-33].

According with the immune panorama above described, in malaria, as result of the prime infection, it would be expected that a population of highly specific memory B cells should be produced, so that when a new host-parasite interaction take place, and the parasite bears the same antigen, the memory cells would be awaken to generate an immune response to eliminate the parasites. The experience dictates that an individual may develop a malaria infection (symptomatic or not) whenever the parasites that reach that individual expresses antigenic variants different to that of the previous infection. In that case, the B cells of that individual have to repeat all the processes of genomic rearrangements each time the individual enters in contact with a parasite possessing a new variety of antigens, giving opportunity for the B cells to produce chromosomal translocations and transform B cells into malignancy as much as the intensity of parasite contacts increase.

A person living in malaria holoendemic areas, in Africa, has the chance of receiving and infective bite once a day or a week and each infected mosquito inoculates from 10 to 100 sporozoites per bite. In nature the human beings do not build a protective immune response against sporozoites, so each time an infected mosquito bites, invasion of the liver cells takes place. This amount of sporozoites seems to be too small a stimulus to evoke an efficient protective immune response. Experimentally, to produce a protective immune response, about a 1000 mosquito bites are necessary [34].

Persistent transmission of malaria parasites through mosquito bites means a persistent antigenic stimulation of B cells in germinal centers, and a persistent genome breaking and repair, and therefore a constant exposure to the risk of an anomalous repair and c-myc/IgH translocation and c-myc activation.

With the analysis done, it seems reasonable to propose that neither malaria parasites nor EBV are the oncogenes per se, but that the neoplasm arises secondary to the host's immune response to a persistent and chronic antigenic stimulation. That immune stimulation may come from malaria parasites, from EBV, from a joint stimulation of both, or possibly from any other agent that causes a similar type of constant antigenic stimulation.

In humans, BL arise at the peak of the immune response after 5 to 8 years of persistent stimulation by the malaria parasites and reactivations of EBV infection. Certainly, not all children submitted to this rhythm of constant malaria re-infection develop BL, in fact, just one to five out of 100,000 children will develop BL, which suggests that the immune cells response to malaria differs among the children population and only some few of them respond in a way that permits a possible malignant transformation of their B cells. In the mouse model, BL arose after a state of sterile immunity was acquired and antigenic stimulation persisted, and also after stimulation was done at the peak of an immune response. In the CD1 mice the amount of animals that develop sterile immunity after malaria infection is meager, and BL was unexpectedly observed in one out of 13 neoplasia cases.

## Conclusions

The CD1 mice may develop a neoplasm histopathologically indistinguishable of human Burkitt's lymphoma, if the mouse is in a sterile immune status against malaria parasites and, continues to receive antigenic stimulation with living parasites, as in the model *Plasmodium yoelii yoeli*i-CD1 mouse, here described.

It is the first time that a neoplasia with the histopathological features of human Burkitt's lymphoma is observed in a non-genetically manipulated mouse.

There is a link between immunity to malaria parasites and human Burkitt's lymphoma-like development, in absence of Epstein Barr virus, in this rodent model.

It is therefore proposed, that the immune response of the host against malaria parasites is the genesis of Burkitt's lymphoma, and that parasites and viral agents are just inducers of the host response. In other words, it is a neoplasm made by the host himself. If those neoplasms are produced by ourselves as response to a chronic exposure to parasites, it could be a biological process not only limited to malaria parasites-host interactions, but also to the persistent and chronic interactions of the host immune system with other agents, such as bacteria, fungus, virus and other parasites, with the condition that the host's immune response share the same or a similar construction of the response to malaria, i.e. persistent, constant and chronic stimulation of the immune system with antigenic variants of the same agent's species.

## Competing interests

The authors declare that they have no competing interests

## Authors' contributions

FM: conceived the study and design it, coordinated the work and wrote the manuscript. JG-A: worked out the histopathological diagnoses. EC: carried out PCR and help in autopsies and electron microscopy. LR: performed the electron microscopy study.

All authors read and approved the final manuscript.
